# Decoupling analysis of medical service provision and population health outcomes: a hybrid evaluation and machine learning approach

**DOI:** 10.3389/fpubh.2026.1946647

**Published:** 2026-10-26

**Authors:** Xianxian Wang, Ziying Qi, Lu Yang

**Affiliations:** 1 Jintan Affiliated Hospital of Jiangsu University, Changzhou, China; 2 School of Public Administration and Law, Northeast Agricultural University, Harbin, China; 3 Changzhou Jintan District Traditional Chinese Medicine Hospital, Changzhou, China

**Keywords:** explainable machine learning, medical service provision, non-linear associations, population health outcomes, Tapio decoupling model, XGBoost-SHAP

## Abstract

**Introduction:**

Global healthcare faces a critical challenge where extensive medical resource expansion fails to translate into proportional population health outcomes due to diminishing marginal returns. The aim of this study was to explore the dynamic evolution, regional disparities, and non-linear associations between medical service provision (MSP) and population health outcomes (PHO) across Chinese provinces.

**Methods:**

Based on panel data from 31 Chinese provinces (2012–2024), an integrated CRITIC-Entropy-TOPSIS approach was constructed to measure MSP and PHO levels. The Tapio model and Sankey diagrams characterized decoupling states and transitions, and XGBoost-SHAP framework quantified feature contributions and non-linear associations among the contextual variables.

**Results:**

Nationally, the median MSP score rose from 0.295 to 0.373 with the spatial gap between eastern and western provinces expanded, but the median PHO score decreased from 0.739 to 0.696. The Tapio analysis revealed widespread non-coordination, with 27 provinces locked in negative decoupling (20 strong negative decoupling, 7 expansive negative decoupling). SHAP analysis identified Population Density (PD, mean absolute SHAP = 0.0174), Urbanization Rate (UR, 0.0126), and Government Health Expenditure (GHE, 0.0119) as the three most important predictors of the decoupling index. The dependence and interaction plots showed non-linear relationships within the provincial sample. Higher PD and UR generally corresponded to higher predicted decoupling values, but higher GHE and IPC corresponded to lower predicted values over some observed ranges.

**Discussion:**

MSP–PHO decoupling was common across Chinese provinces. Increases in medical resources did not always coincide with proportional improvements in population health outcomes. Policymakers should improve the conversion of medical resources into health outcomes through fiscal support based on local needs, stronger healthcare cooperation across regions, and coordinated policies on demographic, socioeconomic and environmental conditions.

## 1 Introduction

Currently, global healthcare sector faces a critical challenge where population health improvements significantly lag behind the rapid expansion of medical resources. Over the past two decades, despite sustained increases in health expenditure and infrastructure across major economies, empirical evidence suggests that growth in medical service provision (MSP) does not invariably translate into proportional population health outcomes (PHO) (1–3). The divergence is mainly rooted in diminishing marginal returns, where resource inputs beyond certain thresholds fail to produce commensurate health gains (4). The COVID-19 pandemic further exposed this mismatch, which demonstrate that extensive resource accumulation and high per-capita spending do not inherently ensure health system resilience or superior crisis outcomes, a reality observed across many high-income nations (5, 6). Consequently, the World Health Organization (WHO) and international observers have sought new strategic directions. Under the initiative of Universal Health Coverage (UHC), the global health agenda has shifted from service availability toward the effective coverage of health interventions (7). Within this context, identifying the decoupling status between MSP and PHO is essential for the transition from scale-driven growth model to result-oriented health systems, so that healthcare delivery effectively yields sustained and equitable health improvements under the health-related Sustainable Development Goals (SDGs) (8).

As the world's largest developing economy, China has followed the global trend through rapid expansion of its healthcare system since the 2009 New Medical Reform. Driven by substantial fiscal investment and the Healthy China 2030 strategy, the country has achieved near-universal insurance coverage and significant scaling of MSP (9, 10). However, the conversion of these inputs into PHO shows obvious regional disparities (11). Despite the aggregate growth in medical resources, health outcomes in some regions have fallen behind, where the expansion of high-end clinical infrastructure outpaces improvements in basic health indicators (12). The potential decoupling between supply-side expansion and actual health dividends challenges the sustainability of China's healthcare transformation. Consequently, revealing the provincial-level decoupling status between MSP and PHO is essential to optimize resource allocation in line with the requirements of high-quality development.

The relationship between MSP and PHO can be understood through the health production function, in which healthcare resources constitute an important, but not exclusive, input into the production of health (13). In this context, greater medical investment can expand service capacity and improve health outcomes, but the marginal contribution could diminish as the level of provision increases (4). Additional resources do not necessarily generate proportional health gains when they are not effectively transformed into accessible and high quality services. From the perspective of healthcare resource allocation efficiency, uneven spatial distribution, inefficient utilization and mismatches betweenMSP and PHO can create frictions in the input-outcome conversion process (14–16). Moreover, the social determinants of health framework indicates that population health is jointly shaped by socioeconomic development, demographic conditions, education and environmental quality (17, 18). These conditions influence both health needs and the capacity of regional health systems to convert medical resources into effective health improvements. Accordingly, MSP-PHO decoupling can be explained by three related mechanisms: diminishing marginal returns to additional medical inputs, inefficient resource allocation and utilization, and differences in regional conditions that limit the health gains from medical services.

Existing studies have examined the relationship between MSP and PHO from several methodological perspectives. Data envelopment analysis have been widely used to evaluate the efficiency with which healthcare inputs are converted into service outputs or health-related outcomes (12). Coupling coordination models have also been applied to assess the degree of synergistic development between MSP and related socioeconomic systems (19). At the same time, traditional econometric methods, including fixed effects models and the generalized method of moments, have also been used to examine the relationships between healthcare inputs, service utilization, and population health (20, 21). These approaches have improved the understanding of resource efficiency, regional inequality, and health system coordination. However, three gaps remain. First, efficiency or coordination measurement are often treated as final outcomes, but do not adequately show whether MSP and PHO change at proportionate rates. Second, estimates of average effects do not fully reflect spatiotemporal changes and possible nonlinear relationships in the process of MSP–PHO conversion. Third, recent studies has used machine learning to identify complex and non-linear associations (22), but few of them have combined it with decoupling analysis to examine contextual factors associated with mismatches between medical inputs and health outcomes. The above limitations restrict the identification of when, where, and under what regional conditions MSP–PHO decoupling occurs.

To address these gaps, this study investigates the extent to which improvements in population health outcomes have kept pace with growth in medical service provision using a balanced panel of 31 Chinese provincial-level units from 2012 to 2024. The two systems of MSP and PHO are first measured using the CRITIC–Entropy–TOPSIS method, which combines information from multiple indicators into comparable composite scores (23). The Tapio model is then used to classify the relationship between changes in the two systems across provinces and over time (24). Finally, XGBoost–SHAP is applied to explore the associations of decoupling with regional socioeconomic, demographic, environmental and human capital conditions (22). The analysis starts with measuring MSP and PHO, then reveal changes in the relationship between the two systems and the conditions associated with greater decoupling. The findings of this study help explain the spatial and temporal pattern of MSP–PHO decoupling in China and on the non-linear relationships observed in the provincial data.

## 2 Materials and methods

### 2.1 Data sources

The empirical analysis in this study covers 31 provincial-level administrative units in mainland China from 2012 to 2024 and explore the evolution of MSP and PHO over time. A balanced panel dataset was compiled mainly from the China Statistical Yearbook and the China Health Statistical Yearbook. The 2013 to 2025 editions were used because each edition mainly reports data for the preceding year. Provincial statistical yearbooks were used to check and supplement the national records. Monetary variables were converted to constant prices using provincial price indices to improve comparability over time.

### 2.2 Research methods

#### 2.2.1 CRITIC-entropy-TOPSIS method

This study employs an integrated CRITIC-Entropy-TOPSIS approach to evaluate the development of MSP and PHO accurately. Compared to the traditional entropy weight method that overlooks the conflicting and redundant relationships among indicators, we integrate the CRITIC (Criteria Importance Through Intercriteria Correlation) method, which accounts for both the contrast intensity (standard deviation) and the conflicting character (correlation coefficient) of the indicators. The final evaluation is executed through the TOPSIS (Technique for Order of Preference by Similarity to Ideal Solution) method to determine the relative closeness of each province to the optimal target (24). The calculation process consists of the following steps, as presented in Equations (1) to (8):

(1) Data Normalization

To eliminate the influence of different dimensions and units, the raw data are normalized. For positive indicators:


$$\begin{array}{l} {\text{x}}_{\text{ijt}}=\frac{{\text{X}}_{\text{ijt}}-\text{min}\left({\text{X}}_{\text{jt}}\right)}{\text{max}\left({\text{X}}_{\text{jt}}\right)-\text{min}\left({\text{X}}_{\text{jt}}\right)} \end{array}$$ (1)

For negative indicators:


$$\begin{array}{l} {\text{x}}_{\text{ijt}}=\frac{\text{max}\left({\text{X}}_{\text{jt}}\right)-{\text{X}}_{\text{ijt}}}{\text{max}\left({\text{X}}_{\text{jt}}\right)-\text{min}\left({\text{X}}_{\text{jt}}\right)} \end{array}$$ (2)

where *X*_ijt_ represents the raw value of indicator j for province i in year t, and *x*_ijt_ is the normalized value.

(2) CRITIC Weighting Calculation

The information amount *C*_j_ of each indicator is determined by its standard deviation σ_*j*_ and correlation coefficient *r*_jk_ with other indicators:


$$\begin{array}{l} {\text{C}}_{\text{j}}={\text{σ}}_{\text{j}}\overset{\text{m}}{\underset{\text{k}=1}{\sum }}\left(1-{\text{r}}_{\text{jk}}\right) \end{array}$$ (3)

The CRITIC weight *w*_j2_ is then calculated as:


$$\begin{array}{l} {\text{w}}_{\text{j}1}=\frac{{\text{C}}_{\text{j}}}{\sum_{\text{j}=1}^{\text{m}}{\text{C}}_{\text{j}}} \end{array}$$ (4)

(3) Entropy Weighting Calculation

The information entropy *e*_j_ and the entropy weight *w*_j2_ are calculated based on the probability matrix:


$$\begin{array}{l} {\text{e}}_{\text{j}}=-\frac{1}{\text{ln}\left(\text{n}\times \text{T}\right)}\overset{\text{T}}{\underset{\text{t}=1}{\sum }}\overset{\text{n}}{\underset{\text{i}=1}{\sum }}{\text{p}}_{\text{ijt}}\text{ln}\left({\text{p}}_{\text{ijt}}\right) \end{array}$$ (5)

$$\begin{array}{l} {\text{w}}_{\text{j}2}=\frac{{1-\text{e}}_{\text{j}}}{\sum_{\text{j}=1}^{\text{m}}\left({1-\text{e}}_{\text{j}}\right)} \end{array}$$ (6)

where n is the number of provinces and T is the number of years.

Combined Weight Determination

CRITIC weighting considers indicator contrast and conflict among indicators, while Entropy weighting reflects the dispersion of indicator values. The final weight was calculated as the arithmetic mean of the two normalized weight vectors:


$$\begin{array}{l} {\text{W}}_{\text{j}}=\left({\text{w}}_{\text{j}1}+{\text{w}}_{\text{j}2}\right)/2 \end{array}$$ (7)

(4) TOPSIS Score Evaluation

The weighted normalized decision matrix is constructed as *v*_ijt_ = *x*_ijt_ × *W*_j_. The ideal positive solution (*V*^+^) and negative ideal solution (*V*^−^) are identified. The Euclidean distances from each evaluation unit to the ideal solutions (${\text{D}}_{\text{it}}^{+}$ and ${\text{D}}_{\text{it}}^{-}$) are calculated. Finally, the relative closeness coefficient (*C*_it_) is derived:


$$\begin{array}{l} {\text{C}}_{\text{it}}=\frac{{\text{D}}_{\text{it}}^{-}}{{\text{D}}_{\text{it}}^{+}+{\text{D}}_{\text{it}}^{-}} \end{array}$$ (8)

The value of *C*_it_ ranges between 0 and 1, where a higher score represents a higher development level of MSP or PHO for province i in year t.

#### 2.2.2 Tapio decoupling model

To reveal the dynamic, directional evolution between MSP and PHO, we adopts the Tapio decoupling model. Unlike the conventional OECD decoupling index, which is highly sensitive to the choice of the base year, the Tapio model utilizes the concept of elasticity to reflect the relative changes between variables, which ensure greater stability and longitudinal comparability (25). The decoupling index (DI) between MSP and PHO is calculated using Equation (9):


$$\begin{array}{l} \text{DI}=\frac{\text{\%ΔPHO}}{\text{\%ΔMSP}}=\frac{\left(\text{PH}{\text{O}}_{\text{t}}-\text{PH}{\text{O}}_{0}\right)/\text{PH}{\text{O}}_{0}}{\left(\text{MS}{\text{P}}_{\text{t}}-\text{MS}{\text{P}}_{0}\right)/\text{MS}{\text{P}}_{0}} \end{array}$$ (9)

where %ΔPHO and %ΔMSP represent the percentage changes in PHO and MSP from the base year 0 to the target year t, respectively.

Based on the sign (positive or negative) of *%ΔPHO* and *%ΔMSP*, together with the critical elasticity thresholds of 0.8 and 1.2, the coordination status between the two systems is categorized into eight distinct decoupling states. These states are broadly grouped into three major domains: decoupling, coupling, and negative decoupling (Table 1). Figure 1 illustrates the quadrant distribution of the eight decoupling states within the Tapio decoupling model.

**Table 1:** Decoupling type classification.

Decoupling state	Decoupling type	%ΔMSP	%ΔPHO	DI
Decoupling	Strong decoupling (SD)	>0	<0	(–∞,0)
	Weak decoupling (WD)	>0	>0	(0,0.8)
	Recessive decoupling (RD)	<0	<0	(1.2,+∞)
Coupling	Expansive coupling (EC)	>0	>0	(0.8,1.2)
	Recessive coupling (RC)	<0	<0	(0.8,1.2)
Negative decoupling	Expansive negative decoupling (END)	>0	>0	(1.2,+∞)
	Weak negative decoupling (WND)	<0	<0	(0,0.8)
	Strong negative decoupling (SND)	<0	>0	(–∞,0)

**Figure 1:**
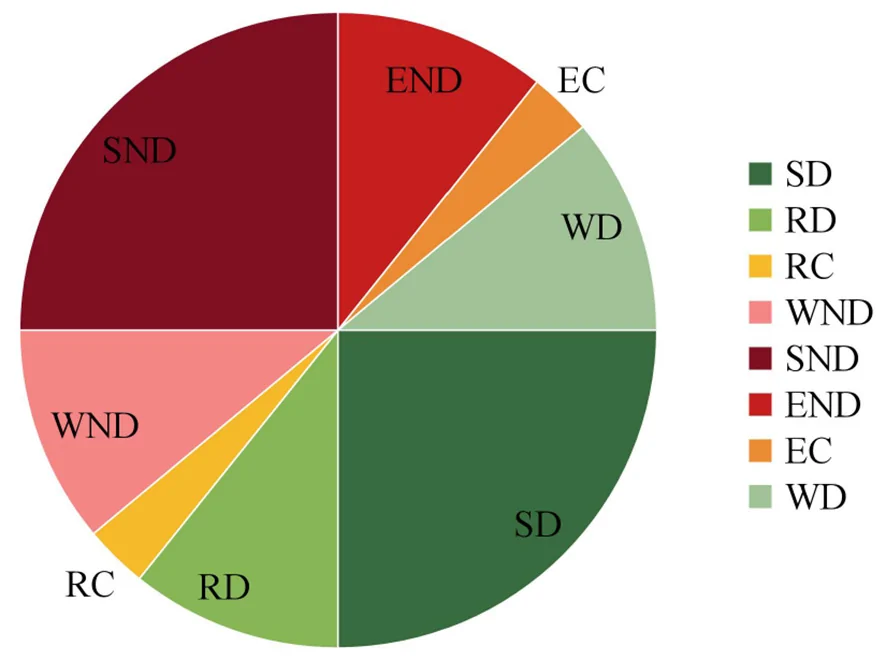
Distribution of eight decoupling states.

#### 2.2.3 The XGBoost-SHAP framework

To explore the contextual factors associated with MSP–PHO decoupling and potential non-linear relationships, this study further applies an interpretable machine learning framework that combines the eXtreme Gradient Boosting (XGBoost) with Shapley Additive exPlanations (SHAP). Traditional regressions methods require non-linear terms and interaction effects to be specified in advance. XGBoost is a tree-based ensemble algorithm (24) that can model non-linear associations and interactions among contextual variables without imposing a predefined functional form.

However, the inherent XGBoost predictions are difficult to interpret directly because the model combines many decision trees. SHAP was used to explain the model output. Based on cooperative game theory, SHAP decomposes each prediction into additive feature attributions and provides locally accurate and consistent attribution (26). The attribution of a specific feature j for a given observation i is calculated using Equation (10):


$$\begin{array}{c} {\text{ϕ}}_{j}\left(i\right)=\underset{\text{S}\subseteq \text{F}\backslash \left\{\text{j}\right\}}{\sum }\frac{|\text{S}|!\left(|\text{F}|-|\text{S}|-1\right)!}{|\text{F}|!}\left[{\text{f}}_{\text{x}}\left(\text{S}\cup \left\{\text{j}\right\}\right)-{\text{f}}_{\text{x}}\left(\text{S}\right)\right] \end{array}$$ (10)

where *F* represents the total set of input features, *S* denotes a subset of features excluding feature j, and *f*_x_(*S*) is the expected output of the model conditioned on the feature subset.

In this study, the calculated DI score serves as the continuous target variable. First, multi-dimensional health determinants are compiled as predictors, with variance inflation factors (VIF) computed to diagnose and mitigate potential multi-collinearity. Second, the dataset is partitioned for model training and optimization via cross-validation to maximize predictive accuracy while preventing overfitting. Finally, the trained model is wrapped with the SHAP tree explainer, SHAP summary plots are used to rank the relative contribution of the predictors to model estimates, while dependence and interaction plots are used to describe non-linear patterns within the observed sample.

In addition, Random Forest and LightGBM were used to check the consistency of key predictor rankings across models. Both models used the same training and test samples as XGBoost. Hyperparameters were selected through five-fold cross-validation on the training sample, and predictive performance was evaluated on the test sample. Since the models used different measures of feature importance, the comparison focused primarily on predictor rankings rather than importance values.

### 2.3 Indicator selection

Indicator selection drew on the WHO–UNICEF Primary Health Care Measurement Framework and China's Healthy China 2030 Planning Outline. The former links health system inputs and service delivery with health outcomes, while the latter emphasizes an integrated service system, equitable allocation of medical resources and health protection across the life course (WHO and UNICEF, 2022; Central Committee of the Communist Party of China and State Council of the People's Republic of China, 2016). Based on this background, the 29 indicators were selected from yearbook series that were available for all provinces and could be followed consistently from 2012 to 2024. Earlier provincial studies have likewise described medical service provision in terms of health workforce, financial investment, institutional capacity and service provision (27).

The MSP system contains 15 indicators in five domains: medical facilities, health workforce, health financing, service activity and performance, and financial burden (Table 2). Hospitals, maternal and child health institutions, public health institutions, and beds describe the physical basis of service provision. Licensed physicians, registered nurses, and pharmacists represent the workforce available to deliver care. Institutional income and expenditure, together with per capita health expenditure, reflect the financial resources supporting healthcare delivery. Outpatient visits and hospital admissions indicate the volume of services actually provided, while emergency case fatality and inpatient mortality offer limited signals of service performance. The share of healthcare expenditure in household consumption was retained to reflect the financial burden faced by service users. These indicators describe changes in provincial healthcare capacity and service activity.

**Table 2:** Construction of the evaluation index system for MSP.

Dimensions	Elements	Unit	Direction	Weight
Medical facilities	Number of Grade A Tertiary hospitals	Units	Positive	0.071
	Number of primary-level M&H institutions	Units	Positive	0.095
	Professional public health institutions	Units	Positive	0.046
	Number of beds in M&H institutions	Beds	Positive	0.087
Medical human resources	Number of licensed physicians	Persons	Positive	0.085
	Number of registered nurses	Persons	Positive	0.092
	Number of pharmacists	Persons	Positive	0.107
Medical revenue & expenditure	Income of medical and health institutions	100M RMB	Positive	0.067
	Costs of medical and health institutions	100M RMB	Negative	0.008
Medical services	Number of outpatient visits	10k	Positive	0.112
	Emergency case fatality rate	%	Negative	0.045
	Number of hospital admissions	Persons	Positive	0.096
	Inpatient mortality rate	%	Negative	0.013
Medical coverage	Per capita health expenditure	RMB	Positive	0.046
	Share of healthcare in household expenditure	%	Negative	0.031

PHO was measured with 14 indicators covering overall health, maternal health, and child health (Table 3). Life expectancy, general mortality, and the incidence and mortality of notifiable infectious diseases describe broad population health conditions. Prenatal examination, systematic maternal management, postpartum visits, hospital delivery, and maternal mortality capture health protection during pregnancy and childbirth. Neonatal visits, child health-management rates, perinatal mortality, and underweight prevalence reflect health conditions and preventive care during early life. This grouping follows the outcome domains used in recent provincial research on public health resource allocation and is compatible with routine health monitoring in China (28).

**Table 3:** Construction of the evaluation index system for PHO.

Dimensions	Elements	Unit	Direction	Weight
Overall health	Incidence rate of Class A & B notifiable infectious diseases	1/100,000	Negative	0.116
	Mortality rate of Class A & B notifiable infectious diseases	1/100,000	Negative	0.142
	Population mortality rate	‰	Negative	0.112
	Average life expectancy	Years	Positive	0.034
Maternal health	Systematic management rate	%	Positive	0.029
	Postpartum visit rate	%	Positive	0.064
	Prenatal examination rate	%	Positive	0.043
	Maternal mortality rate	‰	Negative	0.085
	Hospital delivery rate	%	Positive	0.014
Child health	Neonatal visit rate	%	Positive	0.013
	Systematic management rate for children under 3	%	Positive	0.096
	Health care management rate for children under 7	%	Positive	0.083
	Perinatal mortality rate	‰	Negative	0.062
	Prevalence of underweight in children under 5	%	Negative	0.106

### 2.4 Contextual factors selection

To explore the associations between contextual variables and MSP–PHO decoupling, this study draws upon the WHO's Social Determinants of Health (SDH) framework for variable selection (29). The social determinants of the SDH framework relates population health not only to healthcare resources, but also to socioeconomic, demographic, environmental, and human capital conditions. Consequently, expansion of healthcare infrastructure alone does not cover the broader conditions related to population health. Based on the SDH framework, six variables were selected from four dimensions: socioeconomic context (GDP, GHE), demographic pressure (UR, PD), environmental regulation (IPC), and human capital (AYE). The variables are used to examine contextual associations with MSP–PHO decoupling (Table 4).

**Table 4:** Contextual variables selection under the SDH framework.

Dimension	Variable (Abbr.)	Unit	Reference
Socioeconomic	Gross Domestic Product (GDP)	100M RMB	(30–32)
	Government Healthcare Expenditure (GHE)	10K RMB	
Demographic	Urbanization Rate (UR)	%	(33, 34)
	Population Density (PD)	Persons/km^2^	
Environment	Investment in Pollution Control (IPC)	10K RMB	(35, 36)
Human capital	Average Years of Education (AYE)	Years	(37, 38)

The above six contextual variables describe provincial conditions related to variation in MSP–PHO decoupling. GDP and government health expenditure represent economic and fiscal capacity. Greater economic and fiscal resources can support medical infrastructure and service provision, but the health outcomes also depend on the allocation and use of available resources. Urbanization rate and population density describe the concentration of population and healthcare demand. Population concentration can support more intensive use of medical resources, but high density can also place greater pressure on existing service capacity. Investment in industrial pollution control represents environmental governance and is related to the health pressures arising from industrial pollution. Average years of education represents human capital and is related to health literacy and the use of healthcare services. The non-linear relationships observed in the SHAP analysis provide possible explanations for provincial differences in decoupling. The fitted reference points do not establish causal mechanisms or fixed policy thresholds.

## 3 Empirical results and analysis

### 3.1 Spatial-temporal characteristics of MSP and PHO

The spatiotemporal distribution of MSP across 31 Chinese provinces from 2012 to 2024 is analyzed to provide an overview of the healthcare supply. At the national level, China's MSP showed gradual upward trend during the study period. As shown in the violin plot (Figure 2c), the median MSP increased from about 0.295 in 2012 to 0.373 in 2024, which reflect the continuous expansion of healthcare infrastructure, human resources and financial investment during the implementation of the “Healthy China 2030” strategy. At the same time, the widening shape of the violins over time shows an increasing regional gap between MSP levels, particularly after 2020. The growing difference shows that while the national average improved, the disparity in healthcare supply between well resourced and under-resourced provinces expanded. There is still a big challenge to balanced regional development.

**Figure 2:**
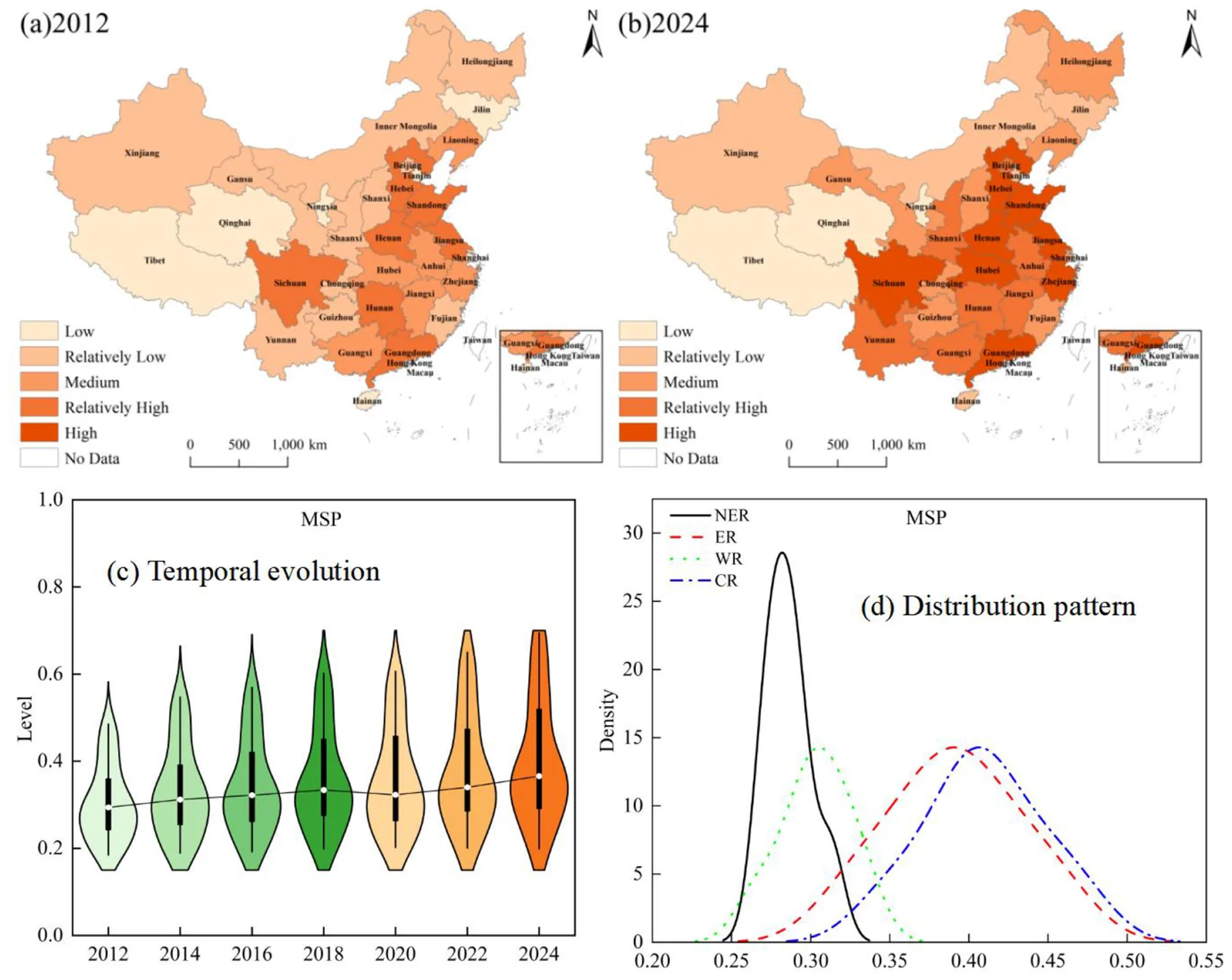
Spatial-temporal characteristics of MSP from 2012 to 2024: **(a)** spatial distribution of MSP in 2012; **(b)** spatial distribution of MSP in 2024; **(c)** temporal evolution of MSP from 2012 to 2024; and **(d)** distribution patterns of MSP across the NER, ER, WR, and CR.

The spatial maps (Figures 2a and 2b) show that regional disparities remain unchanged, with high MSP values concentrated in the eastern coastal provinces and several major inland areas. Provinces with substantial industrial and population bases, such as Shandong, Guangdong, and Henan, consistently maintained or moved into the “High” and “Relatively High” categories throughout the period. By 2024, MSP reached 0.694 in Shandong and 0.673 in Guangdong. High MSP values extended across the Yangtze River Delta, the Bohai Economic Rim, inland hubs like Henan and Sichuan. In contrast, remote peripheral regions, including Tibet, Qinghai and Ningxia remained in the “Low” or “Relatively Low” groups, with values ranging from approximately 0.20 to 0.25.

Furthermore, Kernel Density Estimation (KDE) plot was utilized to analyze the internal changes within different macro-regions (Figure 2d). The density curves for the Central Region (CR) and Eastern Region (ER) are located furthest to the right, which is consistent with the regional averages of 0.468 and 0.455 in 2024. The broader and flatter curves show greater variations among provinces within the two regions.

The curve for the Western Region (WR) is located to the left of the ER and CR distributions, with the main peak concentrated around 0.33. In other words, most western provinces remained at relatively low MSP levels. The Northeast Region (NER) had the lowest overall capacity, increasing from 0.270 to only 0.313 over the study period. The sharp and narrow peak on the far left shows that northeastern provinces were concentrated within a relatively low range.

The spatiotemporal distribution of PHO across 31 Chinese provinces from 2012 to 2024 is analyzed to provide an overview of the health outcomes. At the national level, China's PHO showed slight downward trend and spatial convergence during the study period. As shown in the violin plot (Figure 3c), the median PHO decreased from approximately 0.739 in 2012 to 0.696 in 2024. The marginal reduction reflects the increasing complexity of population health governance under expanding chronic disease burdens and demographic aging, despite continuous healthcare expansion. At the same time, the vertical contraction of the distributions shows an increasing concentration within the 0.70 to 0.85 interval. The shrinking range demonstrates that the absolute performance gaps between regions narrowed, and approached higher-level equilibrium in national baseline health outcomes.

**Figure 3:**
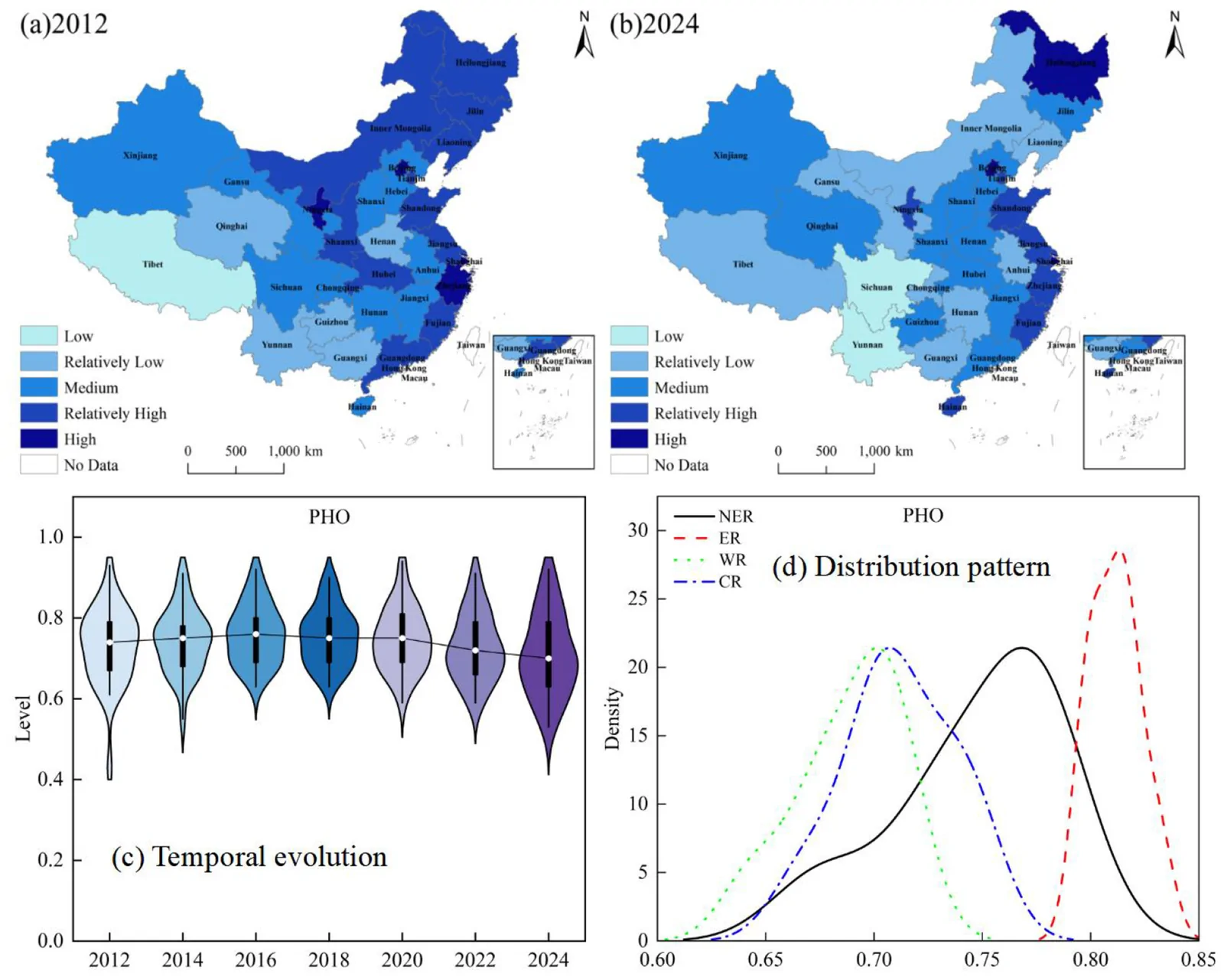
Spatial-temporal characteristics of PHO from 2012 to 2024: **(a)** spatial distribution of PHO in 2012; **(b)** spatial distribution of PHO in 2024; **(c)** temporal evolution of PHO from 2012 to 2024; and **(d)** distribution patterns of PHO across the NER, ER, WR, and CR.

PHO classifications in the spatial maps (Figures 3a and 3b) have broken away from traditional administrative boundaries. In 2012, high PHO values were concentrated in the eastern coastal areas and northern industrial belts, with Beijing (0.930), Shanghai (0.847), and Jilin (0.803) had some of the highest values. By 2024, the spatial distribution had changed significantly. High PHO areas contracted slightly in the south but intensified in the northeast, where Heilongjiang reached 0.919. Meanwhile, key economic centers like Beijing (0.895) and Shanghai (0.880) maintained in the “High” category. In contrast, remote peripheral regions, particularly Tibet, stayed at the bottom of the distribution, with values ranging from 0.430 to 0.627. Furthermore, Sichuan and Yunnan also showed significant declines, and both provinces moved into the “Low” category by 2024.

In the same way, KDE curves was used to compare the distribution of PHO within the four macro-regions (Figure 3d). The regional distributions differed markedly from those of MSP. The curve for the Eastern Region (ER) is located furthest to the right. Its tall and narrow peak corresponds to the high regional average of 0.799 in 2024 and shows relatively small variation among eastern provinces. The Northeast Region (NER) curve occupies the middle-right position, with regional average of 0.735. Its broader and lower peak shows greater variations between rebounding and lagging provinces. The Central Region (CR) and Western Region (WR) distributions lie furthest to the left. Their peaks concentrate near 0.71 and 0.70 respectively, while the regional averages are 0.669 and 0.645. The lower PHO levels in these two regions warrant greater attention in regional health planning.

### 3.2 Decoupling analysis of MSP and PHO

Using the Tapio decoupling model, this section analyzes the geographical changes in the relationship between MSP and PHO over three subperiods and the full study period from 2012 to 2024 (Figure 4). During 2012–2016 (Figure 4a), Expansive Negative Decoupling (END) and Strong Negative Decoupling (SND) were observed in most provinces across the Eastern, Central and Western regions. Weak decoupling (WD) occurred in a few scattered provinces, including Tibet and Qinghai in the west, Shanxi in the central region and Hainan in the east. During 2016–2020 (Figure 4b), the geographical distribution changed, with WD extending westward to form a continuous zone that covered Xinjiang, Qinghai and Tibet. In these provinces, increases in MSP were accompanied by relatively more favorable changes in PHO. The Northeast region showed a mixed distribution, as different provinces in this area falling into distinct decoupling and coupling groups.

**Figure 4:**
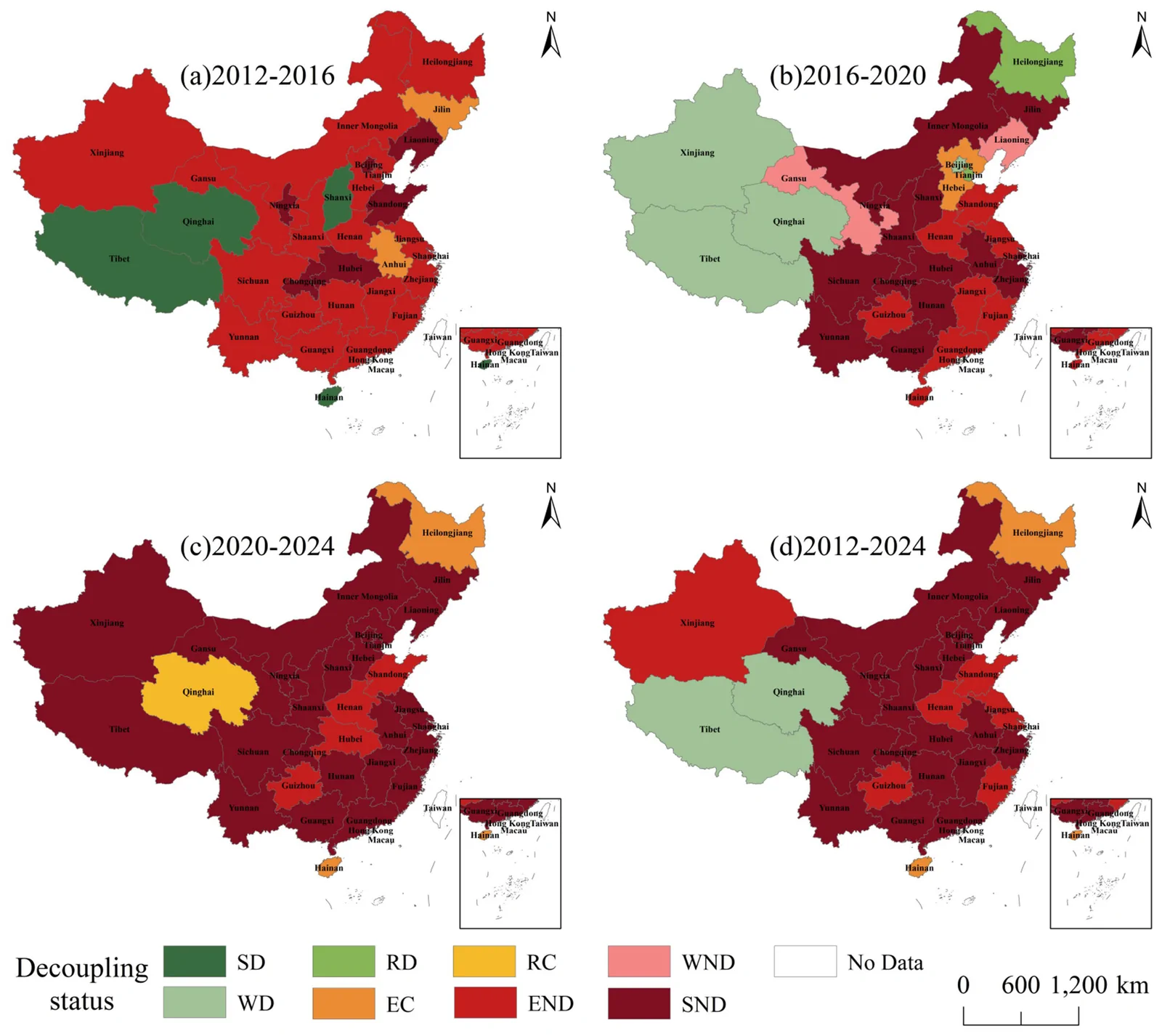
Spatial characteristics of synergy between MSP and PHO during different periods from 2012 to 2024: **(a)** 2012–2016; **(b)** 2016–2020; **(c)** 2020–2024; and **(d)** 2012–2024.

During 2020–2024 (Figure 4c), SND and END categories covered almost all provinces, except for Qinghai (RC), Hainan (EC) and Heilongjiang (EC). Across most provinces, changes in MSP were not matched by proportional improvements in PHO. The full-period map (Figure 4d) shows that the mismatch is not a short term issue, but a long term challenge across most provinces from 2012 to 2024.

The Sankey diagram was used to track the transitions of provinces across different decoupling categories (Figure 5). From 2012 to 2016, most provinces fell into negative decoupling categories. Nineteen provinces were classified as END, including 10 in the ER, six in the CR and three in the WR. Another six provinces fell into the SND category. In most provinces, MSP increased without proportional improvement in PHO. In the next phase of 2016 to 2020, the distribution changed significantly. The number of END provinces dropped from 19 to eight, and four western provinces moved into the more favorable WD category. In these provinces, MSP growth was accompanied by improvement in PHO. But at the same time, the number of SND provinces increased to 14 provinces from the ER, CR and NER. The transitions show improvement in several western provinces but less favorable changes in the relationship between MSP and PHO in many eastern, central and northeastern provinces.

**Figure 5:**
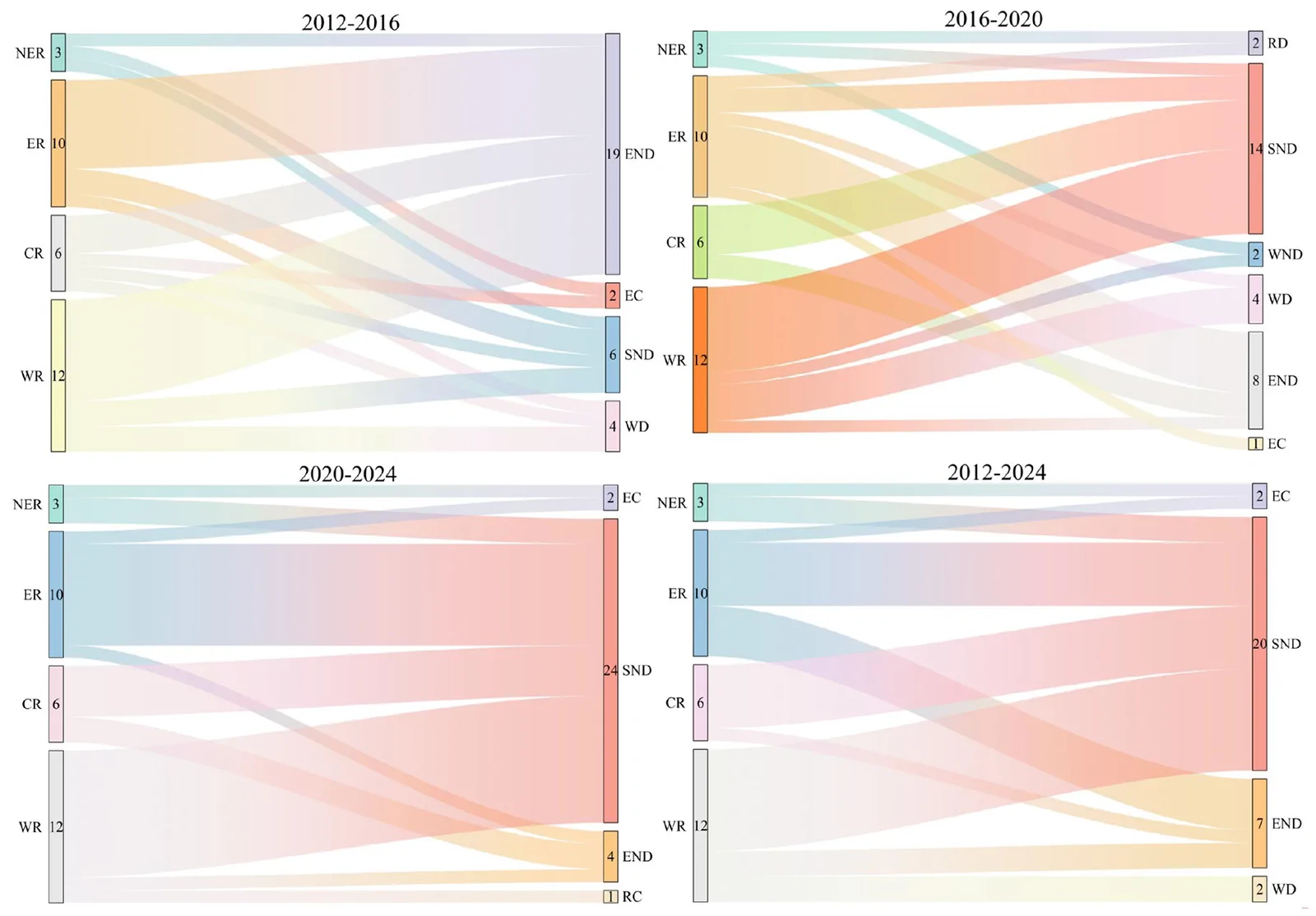
Decoupling relationship between MSP and PHO from 2012 to 2024.

From 2020 to 2024, the provincial flows became concentrated in the negative decoupling categories and the intermediate states largelydisappeared. A total of 24 provinces entered the SND group, and four provinces remained as END. In contrast, only two provinces achieved EC and only one entered RC. These results prove that medical investment outgrew health outcomes in almost all regions. For the full period from 2012 to 2024, 20 provinces stayed in the SND category and seven provinces stayed in the END category. The fact shows that turning resource expansion into health outcomes remains an unresolved issue across China.

### 3.3 Shap-based Interpretability analysis

Before SHAP analysis, the predictive performance of the XGBoost model was evaluated on the test set. As shown in Figure 6, the model obtained an R^2^ of 0.6936 on the test set. The RMSE and MAE were 0.0351 and 0.0228, respectively. Figure 6 compares the predicted and observed decoupling index values. The model was then used to explore the associations between contextual factors and the decoupling index. Turning points in the SHAP plots should be interpreted with caution.

**Figure 6:**
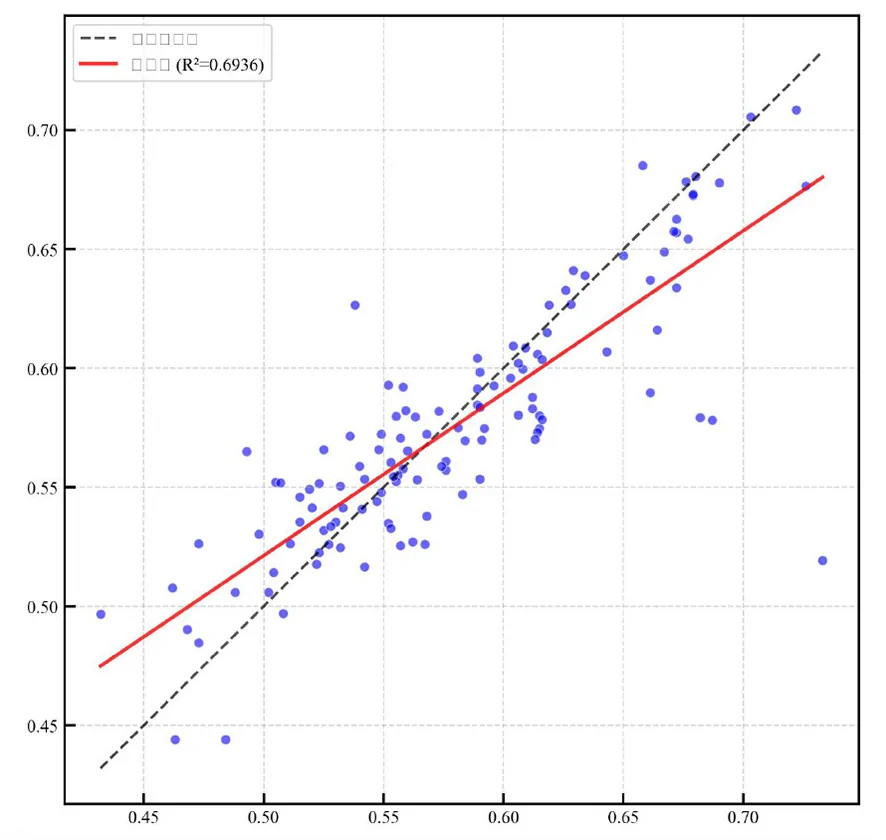
Scatter plot of predicted and actual values of the XGBoost model.

To check the stability of the key predictor rankings, Random Forest and LightGBM were trained using the same training and test samples as XGBoost. Hyperparameters were selected through five-fold cross-validation on the training sample. Both models showed comparable predictive performance on the test sample (Table 5). Predictor rankings were largely consistent across the three models. Population density and urbanization rate ranked first and second, respectively, in all models, and government health expenditure was also among the most important predictors. The rank correlations of the XGBoost–SHAP ranking with the Random Forest and LightGBM rankings were 0.886 and 0.829, respectively.

**Table 5:** Predictive performance and leading predictor rankings across tree-based models.

Model	*R^2^*	RMSE	MAE	Three highest ranked predictors
XGBoost	0.6936	0.0351	0.0228	PD, UR, GHE
Random forest	0.6781	0.0349	0.0252	PD, UR, AYE
LightGBM	0.6524	0.0362	0.028	PD, UR, GHE

Figure 7 presents the SHAP importance ranking and dependence plots. Based on the mean absolute SHAP values, population density ranked first at 0.0174, followed by urbanization rate at 0.0126 and government health expenditure at 0.0119. The three variables also ranked near the top in the Random Forest and LightGBM models. The similar rankings across the three models show that the identification of the leading predictors was not limited to XGBoost. The dependence plots show non-linear relationships within the provincial sample. For population density, with a sample median of 264.41 persons/km^2^, SHAP values generally increased up to around 1,146.22 persons/km^2^ and then leveled off near 0.050 at higher densities. Government health expenditure also showed a non-linear relationship. Below approximately 28.614 billion yuan, SHAP values were mainly positive and varied considerably. At higher expenditure levels, the fitted curve declined and moved into the negative range. For urbanization rate, SHAP values were generally negative below approximately 57%, compared with a sample median of 61%. The values became positive at higher urbanization levels and exceeded 0.04 for some observations above 80%. The numerical turning points describe changes in the fitted curves for the study sample and model.

**Figure 7:**
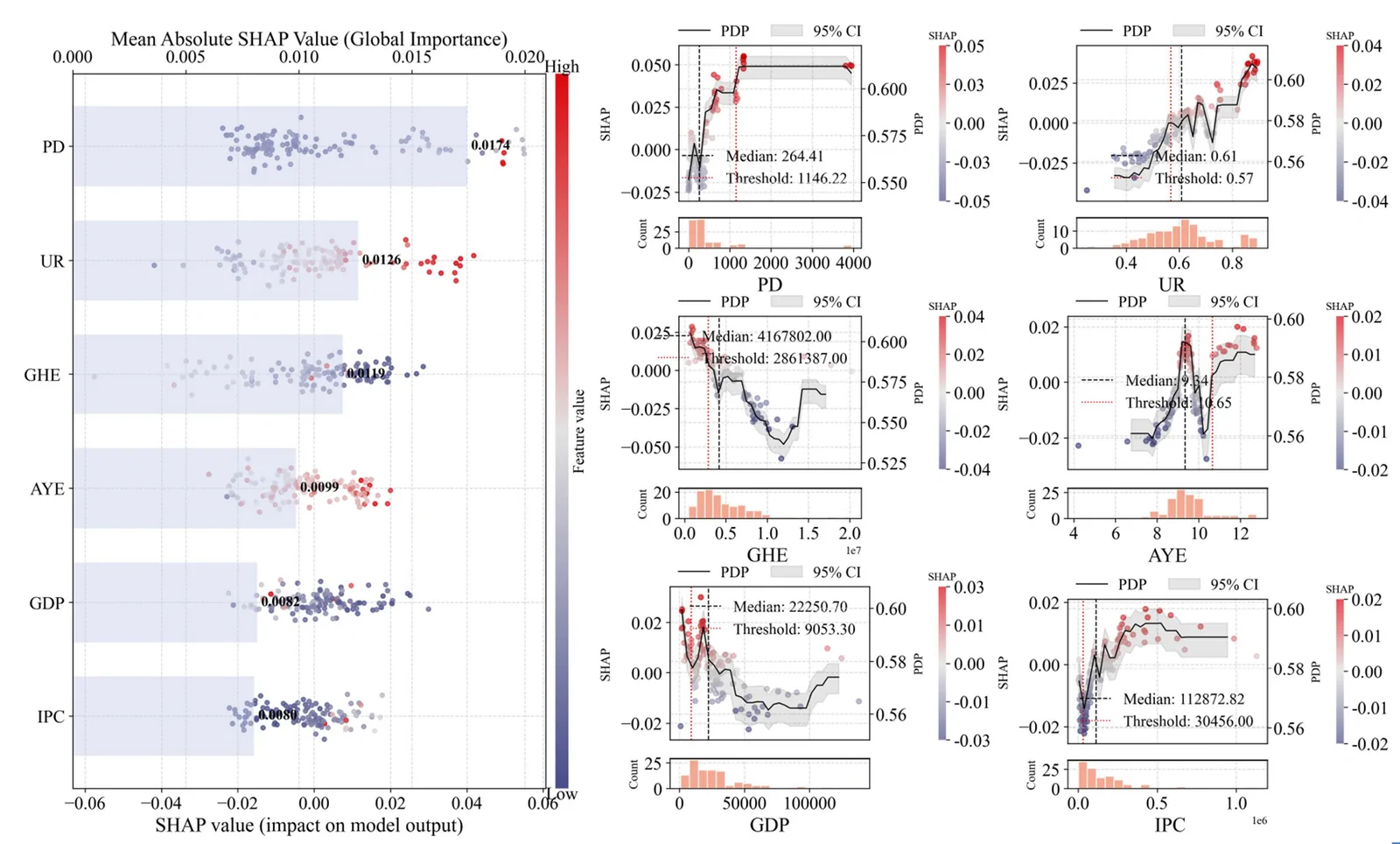
Feature importance and dependency plots based on the SHAP method.

The remaining three factors had lower global importance but showed non-linear relationships in the SHAP dependence plots. For AYE, the fitted curve reached a peak at approximately 10.65 years and then gradually flattened. For IPC, the SHAP value declined after approximately 0.305 billion yuan within the observed range. GDP had higher SHAP values at lower levels and declined after approximately 905.30 billion yuan. The turning points are specific to the study sample and fitted model. The SHAP importance ranking was unchanged under CRITIC and entropy weighting. Government health expenditure, population density and urbanization rate remained the three leading predictors. Under equal weighting, the ranking changed. The relative importance of some predictors was sensitive to the weighting method.

Figure 8 presents four SHAP interaction dependence plots for population density and the other contextual variables. For descriptive visualization, a population density of 288.14 persons/km^2^ was used to divide the observations into lower density and higher density groups. Figure 8a shows the interaction between government health expenditure and population density. In the lower density group, the interaction value changed little as government health expenditure increased. In the higher density group, the interaction value increased over part of the expenditure range. Figure 8b presents the interaction between urbanization rate and population density. The interaction value declined as urbanization increased in the lower density group. For higher density observations, the fitted line rose more rapidly after the urbanization rate reached approximately 0.65. Figure 8c reports the interaction between average years of education and population density. Above approximately 9.67 years, the fitted value for the higher density group first declined and then increased, and the lower density group showed a sharper decline. Figure 8d presents the interaction between pollution control investment and population density. For higher density observations, the interaction value fell below zero when investment exceeded approximately 2.651 billion yuan.

**Figure 8:**
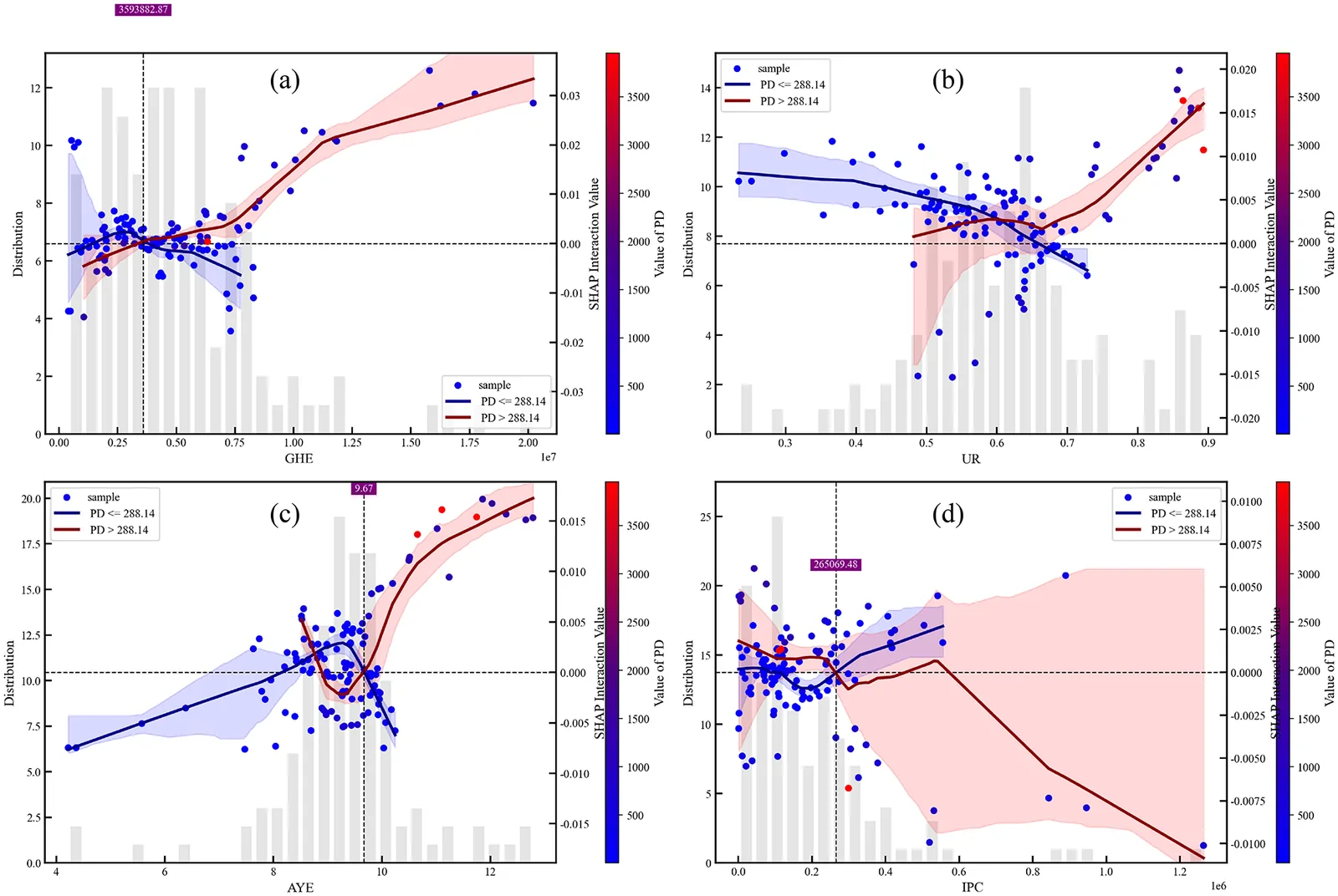
SHAP interaction dependence plot between variables.

## 4 Discussion

### 4.1 Analysis of the synergistic evolution of MSP and PHO

The MSP composite index increased from 2012 to 2024. The index summarizes changes in the measured indicators of medical resources, financing and service activity during the study period. The national median level of MSP increased from 0.295 to 0.373, driven by the expansion of healthcare infrastructure, hospital beds, and medical personnel. However, the Population Health Outcomes (PHO) experienced a slight downward trend globally, with the median dropping from 0.739 to 0.696, coupled with a structural convergence over the [0.70, 0.85] interval. The mismatch between MSP and PHO restricted the further improvement of the system synergy. Disruptions to routine medical services during the COVID-19 period, including chronic disease management and routine immunization, offer one possible explanation for the change after 2020. The mismatch between the two composite indices shows that increases in measured MSP were not accompanied by proportional changes in PHO during some periods. It can be seen that relying simply on physical scale expansion is difficult to sustain, and synergistic development must shift from resource-driven to efficiency-driven. Especially in the post-pandemic era, the decoupling analysis reminds policymakers to break through the single medical supply model and shift to a more resilient life-cycle health management.

### 4.2 Spatial divergence and the ubiquity of MSP and PHO decoupling

Spatial analysis shows obvious regional differences in healthcare supply and health outcomes, but the two systems changed differently over time. Provincial differences in MSP widened, whereas the distribution of PHO became more concentrated. The violin plots and KDE curves show relatively high MSP levels in eastern coastal areas and major inland hubs. Shandong and Guangdong reached 0.694 and 0.673, respectively, by 2024, and Henan also remained among the provinces with high MSP levels. Beijing and Shanghai maintained high PHO values of 0.895 and 0.880. In contrast, Tibet, Qinghai and Ningxia, remained in the low MSP categories, and Tibet also recorded relatively low PHO values. The measured medical resource base increased in western and northeastern regions, but some provinces continued to show less favorable MSP–PHO decoupling classifications. The Sankey diagram shows differences in the transitions of decoupling classifications across regions. Over the twelve study years, 20 provinces stayed locked in the Strong Negative Decoupling (SND) category and seven stayed in the Expansive Negative Decoupling (END) category. The limited movement between decoupling categories shows that regional differences in the relationship between MSP and PHO remained during the study period.

### 4.3 Non-linear associations of contextual factors with MSP–PHO decoupling

Population density (PD), urbanization rate (UR), and government health expenditure (GHE) were the most important factors associated with the predicted MSP–PHO decoupling. PD and UR ranked first and second, respectively, in XGBoost, Random Forest, and LightGBM, which supports the consistency of key predictor identification across models. Higher PD was generally associated with higher predicted decoupling values. UR showed a similar relationship at higher observed levels. The results indicate that population concentration and urban development are closely related to provincial differences in MSP–PHO decoupling. Meeting the demand for outpatient care, hospital services, and public health provision remains an important consideration in densely populated provinces. Coordination of medical services with population mobility and changing health needs also deserves attention during urban development. GHE showed a non-monotonic association with the predicted decoupling, with higher expenditure associated with lower predicted values within certain ranges. The findings support attention to the allocation and use of fiscal resources alongside the scale of spending. Environmental conditions, educational attainment, and economic development were additional factors associated with decoupling. Overall, the results support policies that consider provincial conditions when seeking to improve the conversion of MSP into PHO.

### 4.4 Policy implications for promoting universal health equity

Based on the above discussion, this study proposes the following policy implications. First, a strategic transformation from “resource expansion” to “efficiency improvement” must be achieved. In provinces with high population density, further expansion of physical resources is not necessarily sufficient to improve health outcomes. Policymakers should develop digital healthcare and integrated medical communities to improve service coordination, strengthen primary care capacity and improve service coordination and the delivery of care in densely populated areas. Second, fiscal support should consider local health needs and the capacity of health systems to convert funding into effective services. The observed association between government health expenditure and decoupling suggests that additional funding alone does not necessarily produce comparable health outcomes across provinces. In western and northeastern provinces, Fiscal policy should prioritize primary care, health workforce stability, and the availability of essential public health services. Third, health system planning in densely populated provinces should be coordinated with urban and environmental policies. The interaction analysis shows a stronger association between urbanization and MSP–PHO decoupling in areas with greater population pressure. Higher investment in pollution control was associated with lower predicted decoupling values in some high-density observations. Densely populated provinces can coordinate healthcare investment with environmental improvement, preventive services and primary care development. Specific measures should reflect local needs and implementation capacity.

### 4.5 Limitations and future research

This study has several limitations. First, our analysis relies on annual provincial yearbook data. The MSP system measures the resource base, financing, and service activity of provincial health systems, whereas the PHO system is based on routinely reported health outcomes. Neither system captures several potentially relevant dimensions of the MSP–PHO relationship, including timeliness and quality of care, mental health, functional health, and self-reported well being. Because both systems are derived from multiple indicators, measurement uncertainty in the underlying yearbook data may propagate to the composite scores and subsequent analyses. Second, although the leading predictors ranked similarly across XGBoost, Random Forest, and LightGBM, the SHAP dependence and interaction patterns were estimated from the 2012–2024 provincial panel. The turning points in these curves should be interpreted as empirical reference points within the study sample, and their locations may differ across periods, samples, or model specifications. In addition, the principal SHAP importance patterns were consistent under the CRITIC-only and entropy-only weighting methods but were more sensitive under equal weighting. The machine-learning findings should be interpreted as specification-conditional contextual associations rather than weight-invariant or causal effects. Finally, provincial averages do not adequately reflect within-province differences, particularly among large cities, counties, and rural areas. Future research could combine yearbook data with facility records, population surveys, and data at finer spatial scales to examine the consistency of the observed relationships across settings and periods.

## Data Availability

The original contributions presented in the study are included in the article/supplementary material, further inquiries can be directed to the corresponding author.
